# Effects of Pretreatment Exposure to Dental Practice Using a Smartphone Dental Simulation Game on Children’s Pain and Anxiety: A Preliminary Double-Blind Randomized Clinical Trial

**Published:** 2018-07

**Authors:** Razieh Meshki, Leila Basir, Fateme Alidadi, Azam Behbudi, Vahid Rakhshan

**Affiliations:** 1 Assistant Professor, Department of Pediatric Dentistry, School of Dental Medicine, Ahvaz Jundishapur University of Medical Sciences, Ahvaz, Iran; 2 Dentist in Private Practice, Ahvaz, Iran; 3 Pediatric Dentistry Resident, Department of Pediatric Dentistry, School of Dental Medicine, Ahvaz Jundishapur University of Medical Sciences, Ahvaz, Iran; 4 PhD Student, Department of Cognitive Neuroscience, Institute for Cognitive Sciences, Tehran, Iran

**Keywords:** Anxiety, Pain, Pediatric Dentistry, Computer Simulation

## Abstract

**Objectives:**

Studies on modeling a pre-exposure technique for the prevention of anxiety in children are rare, and there is no study on interactive modeling using computer games. We assessed the effect of playing a dental simulation game before operation on pain and anxiety in 4- to 7-year-old children during their first dental treatment session.

**Materials and Methods:**

In this double-blind randomized clinical trial, 50 children needing unilateral pulpotomy and placement of stainless-steel crowns (SSC) on mandibular primary first molars were enrolled and randomly divided into experimental (a simulation game) and control (no intervention) groups. The experimental group played the game twice a day for two weeks before the scheduled visit. At the dental session, their pre- and post-operative pains were recorded using the Wong-Baker Facial Rating Scale (W-BFRS). Also, heart rate (HR; as an indicator of anxiety) was measured using a finger pulse oximeter at six treatment stages: (1) baseline (at the initial session, two weeks before treatment) and (2–6) during different stages of treatment. Effects of playing the simulation on pain and HR were analyzed using t-test and repeated-measures two-way analysis of covariance (ANCOVA).

**Results:**

Game playing significantly reduced the HR (P=0.031). The interaction of playing with the treatment period was also significant (P=0.004). When the groups were compared in each of the six time points, the experimental group showed reduced HR during anesthetic injection and cavity preparation using a high-speed handpiece (P<0.003).

**Conclusions:**

Based on the results, playing certain dental simulation games before the first dental visit might reduce the anxiety felt during anesthetic injections and drilling.

## INTRODUCTION

Pain and anxiety experienced during dental procedures are common (about 40% of patients might experience dental anxiety, while 20% have a dental phobia) and are among the main concerns in dentistry as pain and anxiety can prevent patients from continuing treatment or seeking it in the first place [1–8]. Pain is “an unpleasant subjective sensory and emotional experience associated with potential or actual tissue injury” [9]. Dental anxiety is an unreasonable and excessive negative emotional state, and its physiological manifestations emerge as a response to fear (probably acquired through previous conditioning with traumatic or negative experiences) or as a reaction to the unknown [1,10–16]. It is important to control the behavior of children, gain their cooperation, and create a positive attitude; pain and anxiety can disrupt this process [17,18]. One of the main issues is behavioral resistance in the first meeting, which introduces several stress-trigger factors, such as unfamiliarity, strange sounds and tastes, the need to constantly lie on the dental chair, discomfort and even pain [6,19].

Since dental fear/anxiety is multifactorial, it is difficult to manage with a single therapy and can lead to unsatisfactory results and a waste of time [2]. Therefore, various pharmacological and nonpharmacological methods have been proposed for its management, including sedatives, voice control, physical restraints, trust building, tell-show-do, and distraction using devices such as virtual reality to control children’s behavior during a dental visit [2,19–24].

According to a 2017 Cochrane systematic review, there are few studies on the efficacy of different non-pharmacological interventions for managing dental anxiety in children [25]. One of the methods for preventing anxiety in the first dental session is pretreatment modeling, which is based on the theory that observing and imitating others might shape behaviors; before undergoing any treatments, the child watches the dental treatment performed on another person and becomes conditioned to show a positive response to dental treatment [12,25–28]. Since anxiety is a common response to uncertainty, and being unfamiliar with a new experience can induce anxiety as the default response to uncertainty [10–15], modeling techniques can introduce dental practices to children as safe and friendly operations, and psychologically prepare them beforehand, preventing anxiety (due to an unfamiliar setting) in the first session. This can be done passively through watching others (in educational films, animated movies [29–33], or live modeling [34,35]) undergoing dental treatments [15,29,34,36], or it can be done in an interactive fashion during playing simulation video games.

Studies on modeling are scarce [25], and studies regarding computer technologies used for anxiety reduction have focused on distraction during the treatment rather than psychological preparation before the dental session [14]. Since smartphones have become very common and technologically very advanced, they can be equipped with proper simulation games to act as a new, convenient, and economical way to reduce dental anxiety in children needing dental treatments. Since studies on modeling are rare [25], and there is no study on playing simulation games using smartphones (or to our knowledge, using any other digital devices) for pre-exposure to dental practice, this preliminary study was conducted to examine the effect of playing a smartphone dental simulation game on pain and anxiety during the first session of dental treatment in children aged 4–7 years attending a private clinic in Ahvaz, Iran in 2016.

## MATERIALS AND METHODS

This preliminary double-blind parallel randomized clinical trial (RCT) was performed on patients referred to a private clinic. A total of 50 children aged 4–7 years who needed pulpotomy treatment of the mandibular first primary molar followed by placement of a stainless steel crown (SSC) were included in the study. The sample size was predetermined subjectively after reviewing the literature and consulting a statistician. As the inclusion criteria, the patients needed to be fully collaborative in the initial examination and needed at least one elective operation in the mandible. None of the included children had a history of dental treatment, hospitalization, or invasive medical treatments. All included children had to be fluent in Farsi (Persian). As the exclusion criteria, children who needed parental care during treatment or those who needed to use the avoidance behavior control methods were excluded. Children with any systemic or mental diseases were excluded. Moreover, children who had been initially included but needed more than one anesthetic cartridge later during the study were replaced with new subjects. Also, emergency cases and parents who did not have Android smartphones compatible with the game were excluded.

During the initial examination, the purpose and the method of the research were explained to the parents, and a written consent was signed by them. No harms were identified with the intervention introduced in this study. The ethics of the study have been approved by the ethics committee of Ahvaz Jundishapur University of Medical Sciences (IR.AJUMS.REC.1395.11) in accordance with the Declaration of Helsinki (DoH). Children excluded from the study still received a complete routine treatment. The methods were designed and pre-registered at a national registry for RCTs available online (code: IRCT2016120531236N1).

### Randomization and blinding:

Before any intervention, the children were randomly assigned to two groups of 25 controls and 25 experimental subjects (video game as the intervention). The first and every other included subject were assigned to the control group, while the included subjects with even numbers were assigned to the experimental group. The study was double-blind, i.e. the pediatric dentist and the research assistant, who were responsible for data collection, were not aware of the study goal and allocations (game versus control). Also, the children were not aware of the grouping and the goal of the study.

### Intervention:

The simulation game of choice should be friendly, not showing the anesthesia needle/syringe, not showing pain, discomfort, or distress of the simulated patient, do present dental instruments preferably with similar sound effects, be popular (indicated by a high number of downloads) with preferably high average user scores, be free and compatible with most models of Android smartphones. Several Android games were found in various websites, downloaded, and evaluated by both a dentist and an experienced pediatric dentist. Games showing needles or any signs of patient pain/discomfort were excluded. Games with low user scores or small download numbers were excluded as well. The selected game was Crazy Dentist - Fun games 1.0 (www.6677g.com). The game had 8 stages with different tasks such as fantasy versions of dental extraction, cavity preparation, filling, scaling, or brushing. There was no image of blood or syringes. Two weeks before the treatment, the game was installed on the parents’ smartphones in the experimental group. They were asked to let children play the game twice a day (15 minutes each time) for the next two weeks until the treatment schedule. If the child played more or fewer than twice a day or if the child missed some days, he/she was excluded from the study and replaced with a new subject. The exclusion was only from the study, and a proper routine treatment was delivered to that child in any case.

### Dental treatments:

All treatment sessions were held in the evening by a pediatric dentist. Block anesthetic injections were performed with one cartridge of lidocaine (Darou Pakhsh Co., Tehran, Iran) after the dentist had initial conversations with the child and applied a topical anesthetic gel. The treatment duration was 30 to 45 minutes. The treatments included unilateral pulpotomy followed by SSC placement on the mandibular primary first molar. All treatments were done after using the tell-show-do method and by using a bur attached to a high-speed handpiece followed by the use of a low-speed handpiece.

### Assessment of pain and anxiety:

During the initial examination session, the Wong-Baker Facial Rating Scale (W-BFRS) was applied before any intervention or treatment. The W-BFRS method is a method for expressing pain in children, which is shown as a sequence of facial expressions: there are six faces with different moods, and each one is assigned a number from 0 to 5, respectively. The face #0 is a smiley face that indicates no pain, and face #5 is crying, which indicates the ultimate pain in the child. The test was explained to the child before being performed. The child was asked to show his/her feelings in one of the pictures before any treatment [37,38]. At the end of the treatment session and before rewarding the child with a gift, the test was repeated.

In order to assess the child’s anxiety, heart rate (HR) was measured as a simple and reliable physiological factor indicative of changes in the level of children’s fear and anxiety [2]. A finger pulse oximeter (Zyklusmed CMS50DL, Zyklusmed, Hamburg, Germany) was used on the right index finger for this purpose as it is convenient, portable, small, and acceptable for children. HR was measured at six different time points as follows: The first time (baseline) was during the first examination session, before any intervention or treatment, when the child was with the parents. The second measurement was recorded immediately after the child was seated on the dental chair (the parents were not in the room). The third measurement was recorded at the time of anesthetic injection. The fourth and fifth measurements were made during cavity preparation using high-speed and low-speed handpieces, respectively. Afterward, the pulse oximeter was removed, the treatment was finalized, and the suction was removed. The dental chair was reset to the upright (default) position, and the sixth HR measurement was made. Also, the W-BFRS was repeated as the post-treatment pain.

After finishing all the tests, the patient was rewarded and sent to the family.

### Statistical analysis:

The groups were compared in terms of pre- and post-operative pains using Mann-U-Whitney test. Also, pains perceived by boys and girls were compared using Mann-U-Whitney test. The multiple ordinal regression was used to evaluate the effects of gender and playing the simulation game on delta-pain, i.e. the post-operative pain minus the pre-operative pain. The groups were compared in terms of the HR and age using independent-samples t-test. Genders were compared using Chi-Square test. The effects of time and playing the simulation game (plus age and gender) on HR were assessed using repeated-measures two-way analysis of covariance (ANCOVA) in SPSS 25 software program (IBM Co., Armonk, NY, USA). The HRs were compared with the average resting HR of the same age (98 beats/minute for 4–6-year olds, and 91 beats/minute for 6–7-year olds) [39] using one-sample t-test. The level of significance was predetermined as 0.05 for all tests, except for t-test used for pairwise comparisons, for which, the level of significance was adjusted to 0.0083 using the Bonferroni method.

## RESULTS

Before reaching the desired sample size, 63 children needing treatment of the mandibular first primary molar were assessed, and 13 were excluded as two children were uncooperative during the treatment, three had not played the game adequately, one needed the presence of parents, and the rest were excluded due to technical issues such as incompatible cell phones. The average ages of the children in the experimental and control groups were 5.59±0.92 and 5.83±0.98 years, respectively (t-test; P=0.375). There were 13 boys and 12 girls in the experimental group, and 9 boys and 16 girls in the control group (Chi-Square; P=0.197).

Preoperative pains were limited to 0 and 1 only: there were two children with pain=1 in the experimental group, and 5 children with pain=1 in the control group (Mann-U-Whitney; P=0.226). The scores of postoperative pains were equal to 0.56±0.96 (range: 0 to 3) in the experimental group and 0.40±0.64 (range: 0 to 2) in the control group (Mann-U-Whitney; P=0.814). The differences between pre- and post-treatment pains were calculated for each patient and were compared between the groups (Mann-U-Whitney; P=0.297).

The scores of preoperative pains were equal to 0.21±0.42 in girls and 0.05±0.21 in boys (Mann-U-Whitney; P=0.814). The scores of post-treatment pains were equal to 0.64±0.91 in girls and 0.27±0.63 in boys (Mann-U-Whitney; P=0.079). The ordinal regression did not show a significant effect for gender (P=0.209) or for playing the simulation game (P=0.291) on delta-pain.

According to t-test, only the third and fourth HR measurements (corresponding to the injection and using a high-speed handpiece) were significantly lower in the experimental group compared to the control (P<0.003; Table 1). The fifth measurement (a low-speed handpiece) was marginally significantly different between the groups (Table 1).

**Table 1.** Comparison of heart rate (HR; beats/minute) measured at six time points in both groups (Independent-samples t-test)

**Time**	**Group**	**Mean**	**SD**	**SE**	**95% CI**		**Min**	**Max**	**P_reference_**	**P_groups_**
**1 (Baseline)**	**Experimental**	98.64	14.84	2.97	92.51	104.77	70	121	0.1731	0.7009
	**Control**	97.08	13.68	2.74	91.43	102.73	74	121	0.2020	
**2 (Sitting on the dental chair)**	**Experimental**	97.56	13.40	2.68	92.03	103.09	77	118	0.2349	0.1732
	**Control**	104.00	19.04	3.81	96.14	111.86	77	151	0.0170	
**3 (Injection)**	**Experimental**	104.92	13.58	2.72	99.31	110.53	80	132	**0.0007**	**0.0002**
	**Control**	121.20	14.51	2.90	115.21	127.19	99	158	**0.0000**	
**4 (High-speed handpiece)**	**Experimental**	106.76	10.06	2.01	102.61	110.91	86	125	**0.0000**	**0.0022**
	**Control**	118.64	15.33	3.07	112.31	124.97	87	150	**0.0000**	
**5 (Low-speed handpiece)**	**Experimental**	107.24	10.38	2.08	102.96	111.52	86	128	**0.0000**	0.0808
	**Control**	113.52	14.22	2.84	107.65	119.39	87	135	**0.0000**	
**6 (Post-treatment)**	**Experimental**	100.76	13.66	2.73	95.12	106.40	70	125	**0.0339**	0.2835
	**Control**	104.92	13.46	2.69	99.36	110.48	85	127	**0.0007**	

SD=Standard Deviation, SE=Standard Error, CI=Confidence Interval, P_reference_=the P-value of one-sample t-test calculated by comparing the HRs with the average resting HR of children of the same age. For each child, the reference for resting HR was set separately according to his/her own age: 98 beats/minute for 4–6-year olds, and 91 beats/minute for 6–7-year olds [39]. P_groups_=the P-value calculated using independent-samples t-test by comparing the groups (α=0.0083)

The rest of the comparisons did not yield significant differences (P>0.17). One-sample t-test showed increases in HR in both groups compared to the resting HR of the same ages. Repeated-measures two-way ANCOVA detected a significant HR-reducing role for the intervention (P=0.031) and for the interaction of the intervention with time (P=0.004), meaning that the pattern of “changes in anxiety over treatment period” differed in the intervention versus the control groups.

**Fig. 1:**
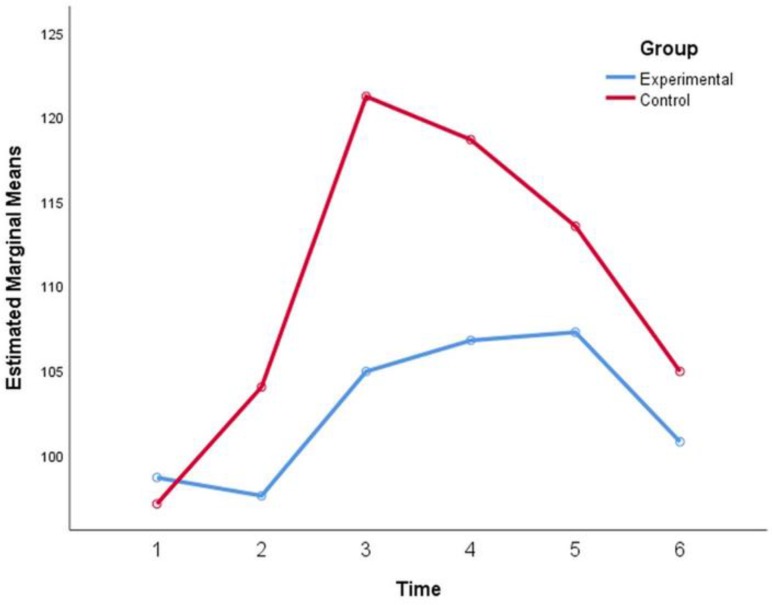
Mean heart rate (HR) values (beats/minute) from the baseline to the end of the clinical session. 1: Baseline; 2: Positioning on the dental chair; 3: Injection; 4: High-speed handpiece usage; 5: Low-speed handpiece usage; 6: End of the session.

The effects of time (P=0.970) and the interaction of time with age and gender (P>0.4) were not significant. The effects of age (P=0.686) and gender (P=0.382) were non-significant as well (Fig. 1).

## DISCUSION

The findings of this study indicated that HR of children did not increase from the reference resting HR until the injection step, after which, it increased in both groups. However, playing the smartphone simulation game before the treatment might partially neutralize this HR increase (by more than 10%) during the injection and drilling with a high-speed handpiece. Playing might not affect the pain. Since we could not find studies on the use of computer simulations for pre-visit psychological preparations, we are limited to discuss the findings in light of more general concepts. Akyuz et al [40] and Milgrom et al [41] found the stages of anesthetic injection and cavity preparation as the ones causing the most anxiety in children; these were in line with our findings, in which, children exhibited reduced anxiety during these critical steps. Our results were in agreement with the findings of most studies on the effects of pretreatment modeling on anxiety using films or live settings as well as the tell-show-do method, which reported significant reductions in anxiety [30–35]. For instance, Al-Namankany et al [33] reported decreased anxiety during examination with a mirror, nasal mask placement, anesthetic injection, and effective tooth extraction without significant reductions in other stages, i.e. in the waiting room, the moment the child entered the room, sitting on the dental chair, and extraction. The difference in anxiety was not significant in the two groups [33]. Farhat-McHayleh et al [34] as well found reduced anxiety in the children exposed to live modeling by their parents compared to those exposed to tell-show-do [34]. This is because the information provided to children for the purpose of psychological preparation creates a cognitive control in them, and thus, reduces the harmful effects of impending anxiety [15]. On the other hand, few studies reported insignificant differences between filmed modeling and the tell-show-do method [29], which could be due to methodological differences, e.g. sample sizes, the quality and duration of the movies, age ranges, cultural backgrounds, and clinical settings. In this study, the pain was not affected by pretreatment playing. The reason might be attributed to numerous factors such as the existence of different pretreatment pains, different expectations for pain, or the fact that almost all pains were zero or slight in both groups, disallowing a vivid contrast. There were no studies on the effect of modeling on perceived pain in pediatric dentistry to compare our results with. However, according to a recent systematic review, pre-surgical psychological preparations might decrease post-surgical pain [42].

Unlike movies and live modeling in which the child is a passive learner, playing computer games can allow an interactive reward-dependent and usually appealing experience for the child and might act as a more attractive option of pre-treatment exposure and modeling to the children of the third millennium [43]. Among computer games, free smartphone applications are much more convenient and easily available, while needing no additional hardware such as virtual reality gears. Therefore, future studies should compare the efficacy of playing games with other methods of anxiety reduction.

This study had some limitations. Although the settings and sample size of this preliminary study sufficed to draw significant results, considering our results, future studies should predetermine the sample size based on power calculations. In addition, it was not possible to monitor the behavior of the subjects during the study course. Furthermore, using a standardized cell phone model (with similar screen sizes, brightness, and sound volumes) for all patients would improve the uniformity of the intervention. However, since in clinical conditions, people do not have similar cell phones and might prefer certain light and sound settings, the present design favored the generalizability. Future studies should design an application specifically for the purpose of pretreatment exposures. However, the current findings showed that even a non-specialized application, if selected carefully, might serve as an appropriate tool for this purpose.

## CONCLUSION

Within the limitations of this preliminary study, it was found, for the first time, that playing certain types of smartphone simulation games for two weeks might reduce dental anxiety (indicated by HR) during anesthetic injection and cavity preparation using a high-speed handpiece.
